# Effectiveness of home-based “egg-suji” diet in management of severe acute malnutrition of Rohingya refugee children

**DOI:** 10.1186/s41043-022-00321-x

**Published:** 2022-11-25

**Authors:** S. K. Roy, Khurshid Jahan, Soofia Khatoon, Nurul Alam, Saria Tasnim, Shahana Parveen, Ambrina Ferdaus, Khadijatul Cubra

**Affiliations:** 1 Bangladesh Breastfeeding Foundation (BBF), Institute of Public Health (IPH), Room #195-201, Mohakhali, Dhaka, 1212 Bangladesh; 2 The International Centre for Diarrheal Disease Research, Bangladesh (icddr’b), Mohakhali, Dhaka, Bangladesh; 3 Community Based Health Care, Mohakhali, Dhaka, Bangladesh

**Keywords:** Severe acute malnutrition (SAM), Egg-suji, Nutrition counseling, Children, Homemade diet, Nutritional status

## Abstract

**Background:**

Prevalence of severe acute malnutrition (SAM) among Rohingya children aged 6–59 months who took shelter in refugee camp in Cox’s Bazar District, Bangladesh, was found to be 7.5%.

**Objective:**

To measure the effectiveness of homemade diet in the management of severe acute malnutrition of Rohingya refugee children.

**Methods:**

In total, 645 SAM children (MUAC < 11.5 cm) aged 6–59 months were selected and fed the homemade diet for 3 months by their caregivers and followed up for next 2 months. Nutrition counseling, demonstration of food preparation and the ingredients of food (rice powder, egg, sugar and oil) were provided to the families for 3 months to cook “egg-suji” diet to feed the children.

**Results:**

The study children were assessed for nutritional status. After intervention, energy intake from diet increased from 455.29 ± 120.9 kcal/day to 609.61 ± 29.5 kcal/day (*P* = 0.001) in 3 months. Frequency of daily food intake improved from 4.89 ± 1.02 to 5.94 ± 0.26 (*P* = 0.001). The body weight of children increased from 6.3 ± 1.04 kg to 9.93 ± 1.35 kg (*P* = 0.001), height increased from 67.93 ± 6.18 cm to 73.86 ± 0.35 (*P* = 0.001) cm, and MUAC improved from 11.14 ± 1.35 cm to 12.89 ± 0.37 cm (*P* = 0.001). HAZ improved from − 3.64 ± 1.35 to − 2.82 ± 1.40 (*P* = 0.001), WHZ improved from − 2.45 ± 1.23 to 1.03 ± 1.17 (*P* = 0.001), WAZ improved from − 3.8 ± 0.61 to − 0.69 ± 0.78, and MUACZ improved from − 3.32 ± 0.49 to 1.8 ± 0.54 (*P* = 0.001) from the beginning to the end of observation. Morbidity was found in 5.12% children in the first month which reduced to 0.15% at the end of follow-up.

**Conclusions:**

Nutritional counseling and supply of food ingredients at refugee camps resulted in complete recovery from severe malnutrition for all children which was sustainable.

## Introduction

An estimated 622,000 Rohingya refugees fled from Myanmar to Cox’s Bazar, Bangladesh, by November 2017. The total number of Rohingya refugees was 835,000, and the estimated proportion of children under 5 years was 29%. The total estimated under-five children was 242,150 in the camp [1].

The Rohingya refugees reached Bangladesh without much assets, and they took shelter in hillside terrain of Cox’s Bazar district [2]. Nutritional assessment in the camps showed a prevalence of 24.3% global acute malnutrition (GAM) and 7.5% severe acute malnutrition (SAM) [3]. The children in Rohingya population in Cox’s Bazar were therefore nutritionally vulnerable.

For young children, severe acute malnutrition (SAM) is a high-risk condition requiring urgent rehabilitation. Referral of SAM children with complications could save the lives of millions of children [4]. On the other hand, socioeconomically privileged children were found to have higher bottle feeding rates in most countries [5].

The role of nutrition education has been proven to significantly improve the nutritional status of moderately malnourished children in Bangladesh within 3 months through improving the child feeding behavior of mothers [6]. Another study showed that an increase in the frequency of complementary feeding was associated with higher weight gain in the nutrition intervention group compared to the control group. Following the intervention, the growth of children was sustained [6, 7]. Presently, many international agencies are promoting Ready to Use Therapeutic Foods (RUTF) for treatment of SAM children [8, 9]. For example, intervention has not been found much successful for sustainability of recovery from SAM in the subcontinent [10]. As a sustainable and cost-effective strategy, nutrition education to the mothers has been reported to be useful to improve and sustain better nutritional status of their children [11]. The home-prepared diets have been tried with success in stable setup, but those have not been tried in emergency situation like the displaced Rohingya children. We conducted this trial to get scientific evidence for the impact feasibility and acceptability of the improved home-based nutrient-dense recipes on recovery from SAM. We tested the hypothesis that providing support with food ingredients and advices on preparation of the home-based nutrient-dense recipes in the refugee camps can cure severe malnutrition in the children with sustainability without food supplement.

## Methods

We identified and enrolled 650, 6–59 months uncomplicated SAM children with MUAC < 11.5 cm. (According to WHO, children who were clinically well without signs of infection or other indication for hospital admission, with a retained appetite passed an appetite test.) Retained appetite is regarded to indicate the absence of severe metabolic disturbance. These subjects are deemed to be most appropriately managed as outpatients. Children who were taking RUTF or refused to cooperate were excluded. Anthropometric measurements such as body weight, length/height and MUAC were taken by trained research assistant to estimate severe wasting (WHZ < − 3 or MUAC < 11.5 cm) [12]. Caregivers of children were explained the purpose of the study before taking consent. Trained research staff worked for 13 groups of Rohingya children in Cox’s Bazar District. Ten groups of children were in Ukhia upazila camp sites, and three groups were in Teknaf upazila camp sites.

**Fig. 1:**
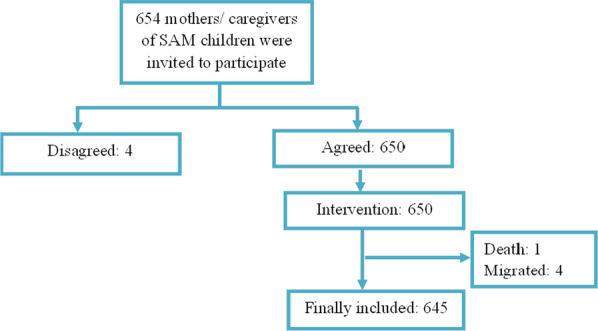
Consort diagram for trial

Nutrition education for caregivers was conducted weekly with demonstration of cooking methods with feeding schedule for improved feeding practice of their children. A total of 645 children were finally enrolled because one child died and four children migrated out during the first week of the study (Fig. 1). The intervention was given for 3 months and then children were followed up for 2 more months.

The refugee families were given shelter by the Govt. of Bangladesh, in small tents having cooking devices and kerosene stoves. Their traditional diet in Myanmar was Sutki-vat (Dried fish and boiled rice). In the camp, they usually consumed rice, dry fish, meat, potato, pulse etc. Sometimes, it has been seen that few families were growing pumpkins around their camp. The refugees also had access to potable water, bathrooms and toilets. Usually five families were allocated to one toilet. They also had tube well facilities where one tube well was used by members of 10–15 tents. Some of them worked as daily worker in the camp for distribution of relief materials. Most of the Rohingya refugees kept contact with Myanmar and continued small business such as selling Burmese cloths, pickles, toys, cream and cosmetics. The Rohingya people received more than adequate amount of relief materials some of which were exchanged for cash. They received additional financial support from the Government of Bangladesh and international NGOs. They appeared reluctant to return to their own country as the situation did not improve to their expected level.

### Nutrition education and nutrient-dense recipe demonstration

Mothers and caretakers of the children were explained the benefits of the ingredients of the nutrient-dense recipe “egg-suji” and how those could improve the nutritional status of their children. Demonstration was given on the preparation of the nutrient-dense recipe. The main components were rice powder, egg, sugar and cooking oil. Nutrition education was provided on the basis of nutrition triangle (food security, disease control, caring practices) as mentioned in an earlier report [6]. Counseling emphasized specific messages for increasing the portion size, frequency of feeding, methods of preparation of the nutrient-dense recipe (egg-suji), storage, hygiene practices, serving to ensure safe food handling and consumption. The selected recipe “egg-suji” was already tested in the improved recipe trial all over Bangladesh where local availability and acceptability were examined. It was adopted from traditionally used local diet and improved in nutrient density in the recipe trial at household level; it was then published in the complementary recipe book of FAO [11]. Egg-suji has been successfully used in hospital setup (Institute of Child and Mother Health-ICMH, Matuail, Narayanganj, Dhaka) for management of children with severe acute malnutrition. At the beginning of the study, Rohingya children were offered two recipes “Khichuri” and “egg-suji,” but the caretakers rejected khichuri as they called it “Gaitta vat” (means a mixed food with rice and lentils which causes diarrhea to children according to their home experience), but the caretakers accepted the ingredients and recipe of egg-suji. Thereafter, the study continued with providing ingredients of egg-suji to respective caretakers.

Ingredients of egg-suji consisting of 7 eggs, 500 g rice powder, 500 ml oil, and 250 g sugar were distributed at weekly intervals for 3 months to the mothers or caregivers of SAM children. At the beginning, egg-suji was made daily by mothers or caregivers with one egg (50 g), two tablespoons of rice powder (suji) (30 g), three teaspoons of sugar (15 g), two teaspoons of oil (10 g) and about 200 ml of water. Mothers used to feed this food to children 2–3 times a day. This amount of egg-suji provided 324 kcal of energy. When acceptability of the diet improved, the amount of oil and suji was increased. In the third month of intervention, egg-suji was improved containing one egg (50 g), five tablespoons of rice powder or suji (75 g), three teaspoons of sugar (15 g), five teaspoons of oil (25 g) and water. This amount of egg-suji provided 614 kcal of energy where carbohydrate provided 46.1% energy, protein provided 10.1% energy and fat/oil provided 44.8% energy (Table 1). The cost of this amount of egg-suji was BDT = 18, equivalent to USD = 0.22 (2018). The mothers were encouraged to continue their family diets for children and continue breast-feeding where applicable.

**Table 1:** Composition of diet in intervention period

Nutrient value	Egg-suji
Energy, Kcal	614.0
Moisture, g	45.8
Protein, g	15.4
Fat, g	30.6
Carbohydrate, g	67.9
Dietary fiber, g	2.9
Ash, g	0.8
Calcium, mg	25.5
Iron, mg	1.6
Magnesium, mg	40.8
Phosphorus, mg	188.9
Potassium, mg	224.3
Sodium, mg	112.5
Zinc, mg	2.8
Copper, mg	0.4
Vitamin A, mcg	82.5
Vitamin D, mcg	1.0
Vitamin E, mg	4.5
Thiamin, mg	0.2
Riboflavin, mg	0.9
Niacin, mg	4.9
Vitamin B6, mg	0.6
Folate, mcg	67.8

Cost: BDT = 18, USD = 0.22 (2018)

The acceptability of egg-suji increased substantially over time, and mothers were very happy to feed their children with the food which they cooked themselves. After 3 months of intervention, food supply was stopped and follow-up was continued for 2 months. Mothers were advised to prepare egg-suji and family foods from their own resources and feed their children. After the intervention was completed, it was advised for adoption of the rehabilitated SAM children in their normal family setup.

### Data collection

Trained research staff identified SAM children (MUAC < 11.5 cm) through anthropometric measurements, collected baseline information with the help of a Rohingya lead person called Majhi (boat man). Food intake of children was estimated by 24-h recall method, and mothers were interviewed for their experiences as well as difficulties if they faced any. Food composition was calculated from the Food Composition Table of Bangladesh [13]. Body weights of the children were measured using an electronic weighing scale (Tanita, HD-314) with a sensitivity of 100 g. For children < 2 years, length was measured using locally made length board with sensitivity to 1 mm in which a measuring tape was fixed between a moveable foot plate and a fixed head board. The mid-upper arm circumference (MUAC) of the children was measured with a MUAC tape (TALC, U.K) with 2 mm divisions.

### Quality control measures

Along with counseling and supply of food ingredients, project staff measured the anthropometric indices, e.g., the body weight, height and MUAC of the children at given intervals. Senior investigators visited the study site two weekly and monthly to monitor activities of staff working at Rohingya camp sites. Five percent of the collected data were re-interviewed with mothers, and anthropometric measurements were reexamined by the investigators for verification within 1 week. Four research representatives from Bangladesh Medical Research Council (BMRC) also visited the Rohingya camps to monitor the activity of the study as independent observer. The study was approved by the Ethical Review Committee of BMRC.

### Statistical analysis

SPSS software (version 20) and WHO Anthro software were used for statistical analysis of the data. Multiple regression was used to measure the relationship of weight gain with determinant variables. Repeated measures analysis of variance (ANOVA) was used to compare among the means of time series data of intervention. Statistical significance was accepted when *P* value was less than 0.05.

## Results

Among the Rohingya children (*n* = 645), girls were more than boys (59.8% vs 40.9%). The mean age of the children was 15.2 ± 6.6 months where 220 (34.1%) children were aged 6 months to < 1 year, 335 (51.9%) were in age 1–2 years and 90 (14%) children were aged more than 2 years. In the baseline, mean ± SD body weight of the study children was 6.3 ± 1.0 kg, height was 67.9 ± 6.2 cm and MUAC was 11.1 ± 1.4 cm (Table 2).

**Table 2:** Baseline characteristics of the SAM children of Rohingya (*n* = 645)

Variables	Mean ± SD
Age (month)	15.22 ± 6.6
Girls/boys (*n*)	386/259
Weight in kg	6.3 ± 1.04
Height in cm	67.93 ± 6.18
MUAC in cm	11.14 ± 1.35
HAZ	− 3.64 ± 1.35
WHZ	− 2.45 ± 1.23
WAZ	− 3.8 ± 0.61
MUACZ	− 3.32 ± 0.49

**Fig. 2:**
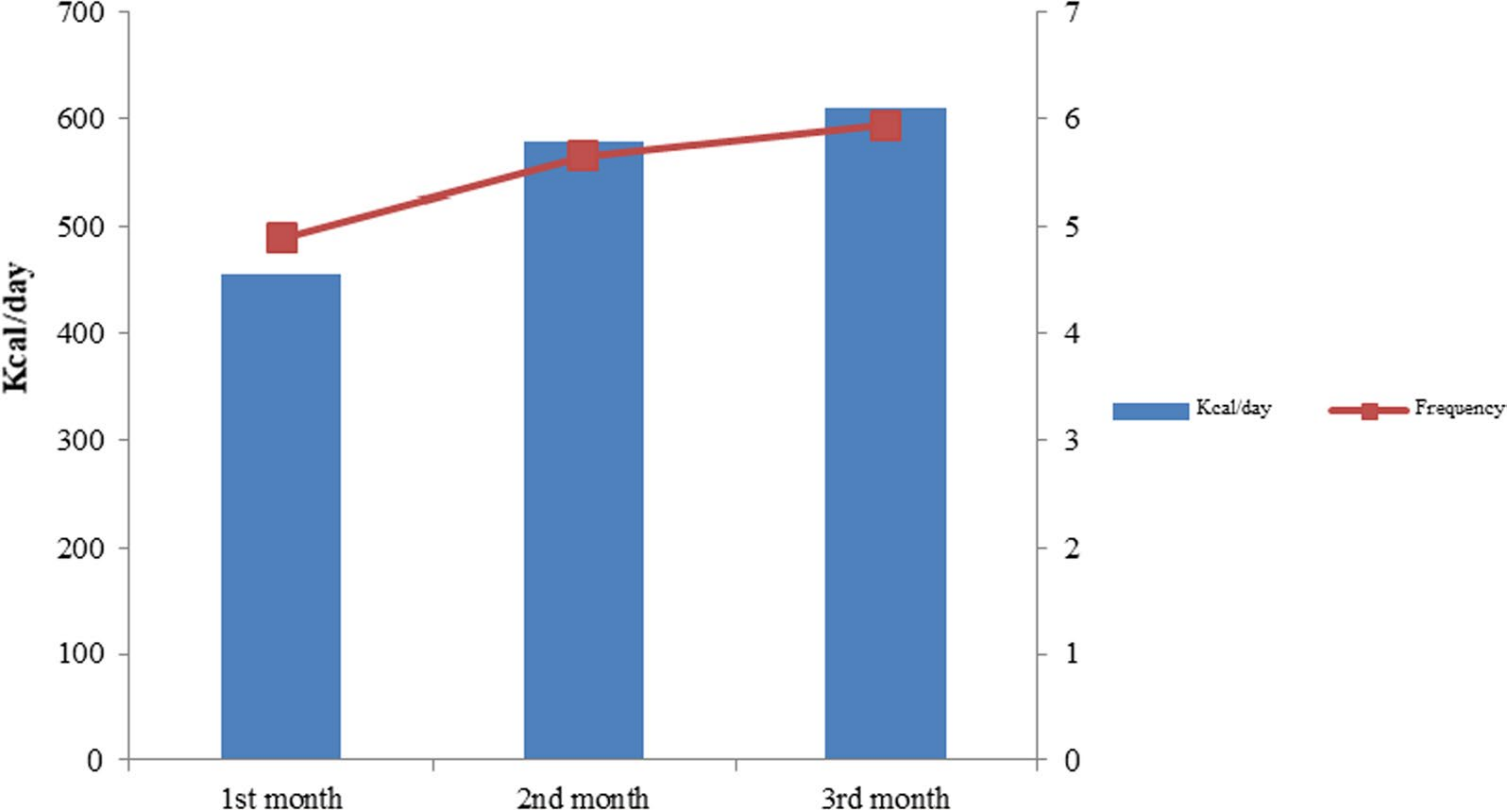
Impact of nutritional intervention on energy intake and frequency of egg-suji (*n* = 645)

The energy intake of children from family food in the baseline was 346.5 ± 22.6 kcal/day. During the first month of intervention, mean energy intake of the children from egg-suji was 455.3 ± 120.9 kcal/day which increased to 578 ± 84.9 kcal/day in the second month and to 609.6 ± 29.5 kcal/day in the third month (*P* = 0.001). In the same time, mean food frequency improved from 4.9 ± 1.0 to 5.7 ± 0.8 and 5.9 ± 0.3 at the end of second and third month of intervention (*P* = 0.001) and the trend continued during the follow-up period (Fig. 2).

Body weight of the study children increased from 6.3 ± 1.0 kg to 9.4 ± 1.3 kg after 3 months of intervention and then to 9.9 ± 1.4 kg at the end of follow-up (*P* = 0.001). The height of the children increased from 67.9 ± 6.2 cm to 72.7 ± 6.2 cm after the intervention and then to 73.9 ± 0.4 cm at the end of follow-up (*P* = 0.001). MUAC of the study children increased from 11.1 ± 1.4 cm to 12.6 ± 6.3 cm at the end of intervention and to 12.8 ± 0.4 cm at the end of follow-up (*P* = 0.001) (Table 3). Mean gain in body weight from baseline to intervention was 3.1 kg, the gain from intervention to the end of follow-up was 0.6 kg, the mean height gain from the baseline to intervention was 4.8 cm and from intervention to end of follow-up was 1.2 cm, and difference in MUAC between baseline and intervention was 1.4 cm and between intervention and end of follow-up was 0.2 cm.

**Table 3:** Changes in mean nutritional status of the SAM Children (*n* = 645) in the study period

Nutritional status (Mean ± SD)	Baseline	After 3 months of intervention	After 2 months of follow-up	*P* value
Weight in kg	6.3 ± 1.0	9.4 ± 1.3	9.9 ± 1.4	0.001
Height in cm	67.9 ± 6.2	72.7 ± 6.2	73.9 ± 0.4	0.001
MUAC in cm	11.1 ± 1.4	12.6 ± 6.3	12.8 ± 0.4	0.001
HAZ	− 3.6 ± 1.4	− 2.9 ± 1.4	− 2.8 ± 1.4	0.001
WHZ	− 2.5 ± 1.2	0.7 ± 1.2	1.0 ± 1.2	0.001
WAZ	− 3.8 ± 0.6	− 0.9 ± 0.8	− 0.7 ± 0.8	0.001
MUACZ	− 3.3 ± 0.5	− 1.9 ± 0.5	1.8 ± 0.5	0.001

Repeated measures ANOVA

Consistent with the increase in weight, height and MUAC, there was a reduction in shortness as their height for age *Z* score (HAZ) changed from − 3.6 ± 1.3 to − 2.9 ± 1.4 after intervention and to − 2.8 ± 1.4 at the end of follow-up (*P* = 0.001). Thinness reduced significantly as weight for height *Z* score (WHZ) reduced from − 2.5 ± 1.2 to 0.7 ± 1.2 after intervention and then to 1.0 ± 1.1 after follow-up (*P* = 0.001). Low body weight reduced as the weight for age *Z* score (WAZ) reduced from − 3.8 ± 0.6 in baseline to − 1.0 ± 0.8 after intervention and to − 0.7 ± 0.8 after follow-up (*P* = 0.001). Mid-upper arm circumference *Z* score (MUACZ) improved from − 3.3 ± 0.5 to − 2.0 ± 0.5 after intervention and then to 1.8 ± 0.6 after follow-up (*P* = 0.001) (Table 3).

In the beginning, 99.1% children were underweight (< − 2 WAZ) in baseline, but the proportion reduced to 6.2% at the end of follow-up. In the beginning, 96.3% children were severely underweight (< − 3 WAZ), but at the end of follow-up, the proportion reduced to 0.5% (Table 4). Initially, 90% children were stunted (< − 2 HAZ) but reduced to 75.4% at the end of follow-up. Proportion of severely stunted (< − 3 HAZ) children in baseline was 70.7% which reduced to 45.3% at the end of follow-up. At the baseline, 65.3% children were wasted (< − 2 WHZ), but the proportion reduced to 0.3% at the end of follow-up. The proportion of severely wasted (< − 3WHZ) children were 30% in baseline but completely eliminated after the intervention. Initially, 100% children were SAM (MUAC < 11.5 cm) in the baseline, but they reduced to 0.0% at the end of follow-up.

**Table 4:** Changes in prevalence of severe acute and moderate malnutrition among the children in the study period (*n* = 645)

Nutritional status	Baseline (%)	After 3 months of intervention (%)	After 2 months of follow-up (%)
WAZ < − 3	96.3	1.1	0.5
WAZ < − 2	99.1	9.6	6.2
HAZ < − 3	70.7	46.4	45.3
HAZ < − 2	90	77.1	75.4
WHZ < − 3	30	0	0
WHZ < − 2	65.3	0.9	0.3

In the first month of intervention, 33 (5.1%) children were found sick and the proportion reduced to 25 (3.9%) in the second month of intervention and their number further reduced to 3 (0.5%) in the third month of intervention. Only 1 child (0.2%) became sick during the 2 months of follow-up period.

**Fig. 3:**
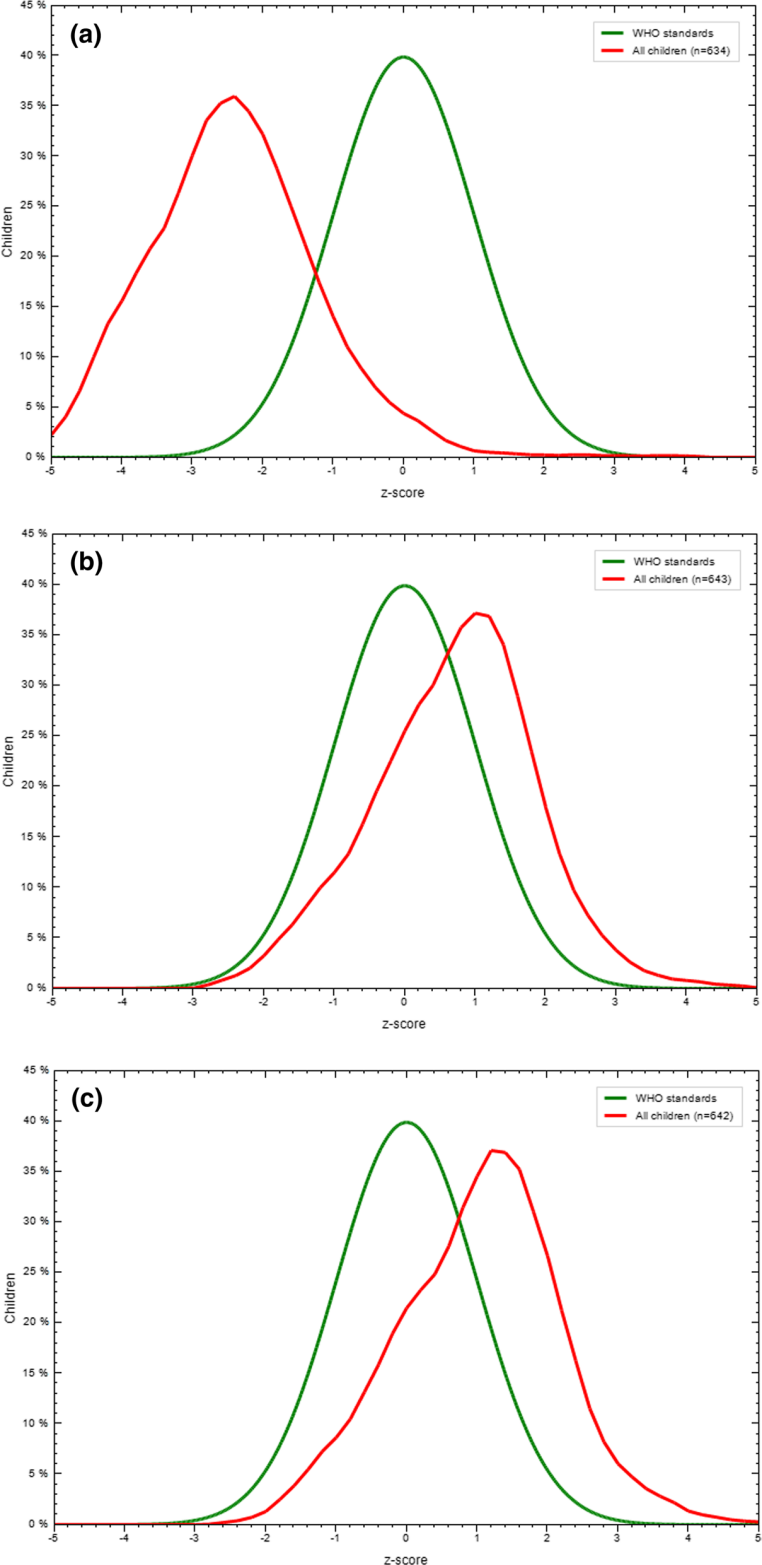
Comparison on weight for height *Z* score with WHO standard by Gaussian curve (I–III). (**a**) Baseline (− 2.45 ± 1.23). (**b**) After 3 months of intervention (0.71 ± 1.16). (**c**) After 2 months of follow-up (1.03 ± 1.17)

The prevalence of SAM reduced from baseline to intervention and from intervention to follow-up (Fig. 3). At the beginning, WHZ distribution of children was left to the WHO standard normal curve. After the intervention, the distribution of WHZ of children moved right to WHO standard Gaussian curve.

Multiple regression for weight gain of children at the end of intervention showed significant association (*P* = 0.001) with initial weight of the children, energy intake from egg-suji and frequency of feeding (Table 5).

**Table 5:** Multiple regression for weight gain of children at the end of intervention

Predictor variable	Slope	SE	B	*P* value
Initial weight of children (kg)	0.15	0.03	0.1	0.001
Energy intake from egg-suji (kcal/day)	0.1	0.004	0.01	0.007
Frequency of egg-suji intake	0.07	0.28	1.04	0.001
Morbidity	0.004	0.4	0.04	0.92

Multiple *R* = 0.23, adjusted R square = 0.05 (*P* = 0.001)

During the follow-up period, from the home food, 86.4% children consumed rice, 32.6% children consumed fish/meat/egg, 25.7% children took pulses, 52.1% children consumed vegetables as well as other foods like rice, dry fish, meat and potato (data not shown).

## Discussion

Our study showed that feeding of homemade recipe with diverse foods by the mothers could improve the nutritional status of the children in 3 months. The food was low in cost which was USD 0.22 (BDT 18) to provide 614 kcal for 6 serving in the whole day. According to our results, if food ingredients are made available, SAM children can be cured at home without commercial foods. During the follow-up period of the study, we did not provide any food support, but we observed that nutritional status of children improved further. The recipe was made by family itself and fed along the family diets.

Initially, the Rohingya mothers were reluctant to accept ingredients of food, but after explaining the role of each ingredient for the recovery from SAM, they began to accept. Demonstration of the nutrient-dense food preparation at home by trained health workers helped the mothers to cook and feed their children with homemade food with change of child feeding behavior. Complete recovery from SAM was a clear demonstration of women’s empowerment that mothers gained new knowledge and skills and were able to cook the right food to cure their children from severe malnutrition.

Recovery from malnutrition required intake of sufficient energy, protein and micronutrients. During the rehabilitation phase, egg-suji provided essential amino acids, energy and micronutrients which were microbiologically safe due to cooking by mothers and scientifically sound for growth of young children. The food was low in cost (BDT 18 for 614 kcal), whereas in Malawi, a study calculated that the food cost of treating SAM with RUTF had monopolized 25% of all child health expenditures to reach only 2% of the child population [14]. A systematic review showed that the total cost of management per SAM child was $203 by giving RUTF in Zambia, whereas in our study the total cost was only $40 being 20% of RUTF management cost [15].

Gain in body weight was faster than gain in height during the rehabilitation period. Stunting is a sign of long-standing failure to thrive mainly affecting the skeletal growth which is dependent on micronutrients like calcium, phosphorus, zinc and vitamin D content of the diet. During the 3 months of intervention, increase in body weight by 57.6% of the initial weight was remarkable and this was significantly correlated with the energy intake. The impact of illness was not significantly associated with gain in body weight because only a very small proportion of children suffered from illness during intervention. The rate of height gain was notable as there was 25.4 percentage point reduction (36%) in stunting. It could be related to the intake of high-quality diet providing essential nutrients.

The composition of this therapeutic diet was close to usual home diet as carbohydrate provided about half of energy, protein provided about one-tenth and fat provided little more than one-third of energy of the diet. A study in India compared acceptability of RUTF (58%) and cereal-legume-based Khichri (77%) where Khichri was seen to be better [16]. The Rohingya children with SAM were in high risk of morbidity and mortality without any support in the emergency situation, so it was unethical to keep these children in a control group without giving any nutritional support by rehabilitation. In addition to continued breast-feeding, mothers were suggested to feed their children 5 times in a day as 3 major meals and 2 snacks [17]. Frequency of feeding is considered as a critical component in child feeding practice as it can ensure increased amount of food intake irrespective of the small size of their stomach. Other studies have reported that when caregivers gave frequent feeding to their children, they were more likely to improve in nutrition at status [6, 18].

Egg-suji has been derived from family snack food for children and adults in Bangladesh. It is also chosen for its convenience of cooking in short time taking 7–10 min and an attractive tasty food normally used and it has high acceptability in Bangladesh [11]. While a sick baby was being fed food for recovery from sick condition, adult family members were seen to be supportive and were not seen to be eager to share a sick babies “egg-suji” food. The ingredients of egg-suji had appropriate nutrients to promote growth of these severely malnourished children. Egg has been considered as a powerhouse of nutrition due to its excellent profile as a nutrient-dense food containing a balanced source of essential amino acids and fatty acids, some minerals and vitamins as well as a number of defensive factors to protect against bacterial and viral infections [19, 20]. Egg contains methionine as limiting amino acid which helps in protein synthesis and formation of enzymes and hormones. Sugar provided direct energy, reduced risk of hypoglycemia and improved taste of diet. Rice powder provided energy, B vitamins and protein; oil improved taste, enriched energy density of the diet, essential fatty acids and reduced the viscosity of diet which helped children to swallow. To improve feeding practices, it was helpful to empower the mothers with knowledge of food ingredients and skills for cooking the diet “egg-suji” [21]. The homemade recipe was convenient and sustainable after the intervention period. The research staff supervised and encouraged the mothers to sustain a good child feeding practice to overcome severe malnutrition.

Frequent morbidity is a barrier against normal growth [22]. Initially, many of our study children suffered from fever, diarrhea, pneumonia, diphtheria, skin disease, etc. Children were referred to attend nearby primary healthcare center within the camps. Along the improvement in nutritional status, morbidity of children gradually reduced indicating improved immunity and better hygiene practices [23]. Further, the systematic review of 14 studies showed very little weight gain by giving RUTF (0.11 g/kg/day) compared to homemade nutrients-dense egg-suji recipe (5.0 g/kg/day) in our study [24].

Studies have indicated that SAM children who are hospitalized suffer from complications such as infections, leukocytosis, imperceptible pulse, pneumonia, septicemia and hypothermia had a high risk of mortality [25]. The Rohingya children were well cared by medical centers and free health centers of the Government of Bangladesh in their camp. It is important to note the extremely low mortality rate of our study children. It appears that the whole of the Rohingya children were well protected as they had an under-five mortality rate of 0.031 per thousand during the study period [25]. Further, the study subjects were selected according to the WHO criteria that there were no immediate reasons for hospitalization. During the  rehabilitation, the parents were always encouraged to seek medical advice for their children in case of illness. However, one of the study children died during the study period.

The results of our study indicate that RUTF or any other commercially made or imported ready-to-eat food containing high fat and high energy (60% from fat) is not required for management of SAM of the children [26]. Other studies also revealed scientific evidence to have better outcome of homemade food over RUTF for SAM treatment [23]. RUTF is extremely expensive compared to egg-suji feeding (USD 5 vs 0.22, i.e., 23 times more expensive) [15]. SAM children are abundant in resource-poor countries of the world; therefore, the cost of treatment is a major concern where other competing needs are not met.

Some limitations of our study need to be mentioned here, such as we faced communication problem with the Rohingya refugees for language but we could overcome it by the help of locally available interpreters. Secondly, our working hour was short during winter due to security problem. Thirdly, when we stopped food supply, the mother appeared to lose interest to speak with us as before. Lastly, we admit that we could not have a control group of similar SAM children as this was not approved ethically in refugee camp situation.

This research used the standard design of intervention, selection of sample, the intervention that combined the supply of food ingredients and demonstrated a nutrient-dense recipe of egg-suji combined with knowledge-based nutrition counseling. The strengths of the study were the appropriate methods for intervention, data collection, quality control, data analysis and interpretation; also, the study results offer unique opportunity to save and improve SAM children at home without misusage of large amount of resources.

## Conclusion

We conclude that family-made local, diverse and nutrient-dense food can completely eliminate and cure SAM children in refugee camps by their own caretakers. The result of this study encourages to apply such simple and cost-effective solution for treatment of uncomplicated SAM children.

## Data Availability

The data sets have been generated and analyzed during the current study, and they are not publicly available due to the organization policy but are available from the corresponding author on reasonable request.
